# Early Psychiatric Consultation Is Associated With Decreased Cost and Length of Stay in the Patient Population at a Level I Trauma Center

**DOI:** 10.7759/cureus.17572

**Published:** 2021-08-30

**Authors:** Marin A Chavez, Jason P Caplan, Curtis A McKnight, Andrew B Schlinkert, Kristina M Chapple, James A Mankin, Jordan V Jacbos, James N Bogert, Hahn Soe-Lin, Jordan A Weinberg

**Affiliations:** 1 Department of Trauma/Acute and General Surgery, St. Joseph's Hospital and Medical Center, Phoenix, USA; 2 Department of Psychiatry, St. Joseph's Hospital and Medical Center, Phoenix, USA; 3 Department of Trauma/Acute and General Surgery, Creighton University School of Medicine, St. Joseph's Hospital and Medical Center, Phoenix, USA

**Keywords:** trauma patient, psychiatric consultation, hospital cost, patient outcomes, level i trauma center

## Abstract

Introduction

Psychiatric illness impacts nearly one-quarter of the US population. Few studies have evaluated the impact of psychiatric illness on in-hospital trauma patient care. In this study, we conducted a retrospective cohort study to evaluate hospital resource utilization for trauma patients with comorbid psychiatric illnesses.

Methodology

Trauma patients admitted to a level I center over a one-year period were included in the study. Patients were categorized into one of three groups: (1) no psychiatric history or in-hospital psychiatric service consultation; (2) psychiatric history but no psychiatric service consultation; and (3) psychiatric service consultation. Time to psychiatric service consultation was calculated and considered early if occurring on the day of or the day following admission. Patient demographics, outcomes, and resource utilization were compared between the three groups.

Results

A total of 1,807 patients were included in the study (n = 1,204, 66.6% no psychiatric condition; n = 508, 28.1% psychiatric condition without in-hospital psychiatric service consultation; and n = 95, 5.3% in-hospital psychiatric service consultation). Patients requiring psychiatric service consultation were the youngest (P < .001), with the highest injury severity (P = .024), the longest hospital length of stay (P < .001), and the highest median hospital cost (P < .001). Early psychiatric service consultation was associated with an average saving in-hospital length of stay of 2.9 days (P = .021) and an average hospital cost saving of $7,525 (P = .046).

Conclusion

One-third of our trauma population had an existing psychiatric diagnosis or required psychiatric service consultation. Resource utilization was higher for patients requiring consultation. Early consultation was associated with a savings of hospital length of stay and cost.

## Introduction

The National Institute of Health (NIH) reported in 2019 that more than 20% of adults in the United States were living with a psychiatric illness [1]. For a high-volume trauma center treating 3,000 patients per year, this translates to nearly two patients with co-occurring psychiatric illness per day or more, given the increased likelihood of trauma patients having a psychiatric diagnosis [2,3]. These patients are at increased risk for adverse outcomes and lengthier hospitalizations, ultimately increasing resource consumption [4-8]. Two studies, published more than 20 years ago, have evaluated hospital costs for trauma patients with psychiatric illness: Zatzick et al. [8] and Lyons et al. [9]. Although Lyons et al. concluded no significant difference in hospital cost, Zatzick et al.'s study suggested psychiatric diagnoses were associated with increased hospital length of stay and cost, and these authors concluded future research should investigate the impact of the early psychiatric diagnosis on outcomes. The purpose of this study was to provide a contemporary evaluation of hospital resource utilization for trauma patients with psychiatric illness versus those without and to explore the impact of timing of psychiatric consultation on hospital-related outcomes and costs.

## Materials and methods

Following approval from our Institutional Review Board, patient admissions to an American College of Surgeons verified Level I trauma center were queried from January 1, 2018 to December 31, 2018. Expired cases and outliers including hospitalizations greater than 30 days and costs greater than $150,000 were excluded. Data collected included patient demographics, injury characteristics, in-hospital specialty consultations, length of stay, and hospital costs. Our cohort was split into three groups: (1) patients with neither a psychiatric diagnosis nor psychiatry service consultation; (2) patients with an existing and managed psychiatric condition but without psychiatry consultation; and (3) patients with psychiatry service consultation. Time to psychiatric consult was evaluated with consultation on the day of or the day following admission considered early and two or more days after admission considered to be a late consult. Comparisons between groups were made using analysis of variance (ANOVA) for age and injury severity score, Pearson chi-square for gender, mechanism of injury, drug use, ICU admission, and mechanical ventilation. Kruskal-Wallis tests were used to compare medians across groups for hospital length of stay, ICU length of stay, ventilation days, and hospital costs. Means for hospital length of stay and costs by self-harm were compared using independent t-tests. Multivariate linear regression models were used to determine the impact of early psychiatric consultation on hospital length of stay and cost. Hedges’ g-statistics were reported as an effect size for independent sample’s t-test and eta-squared values for ANOVA and Kruskal-Wallis tests. Due to differences in discharge processes and reimbursement algorithms by payer, our multivariate models included Medicare/Medicaid patients only. As a result, for the purposes of our models, hospital cost and length of stay were normally distributed and were included as raw units.

## Results

Our cohort consisted of 1,807 patients with a mean age of 50.2 ± 21.5 years. The majority of patients were male (58.3%) with a mechanism of injury of fall (39.8%), followed by motor vehicle accidents (31.4%). Our cohort was categorized into three groups: 1,204 (66.6%) without psychiatric diagnosis or in-hospital psychiatric service consultation, 508 (28.1%) with psychiatric diagnosis but without in-hospital psychiatric service consultation, and 95 (5.3%) who received psychiatric service consultation (Table 1).

**Table 1:** Patient demographics, injury characteristics, and outcomes.

			All (N = 1,807)	No psychiatric condition (n = 1,204, 66.6%)	Psychiatric condition without consultation (n = 508, 28.1%)	Psychiatric consultation (n = 95, 5.3%)	P-value
Age, years			50.2 ± 21.5	52.4 ± 22.4	46.6 ± 18.8	42.2 ± 17.1	< .001
Male sex			1,054 (58.3%)	642 (53.3%)	354 (69.7%)	58 (61.1%)	< .001
Primary insurance							< .001
		Medicaid	702 (38.8%)	386 (32.1%)	261 (51.4%)	55 (57.9%)	
		Medicare	405 (22.4%)	311 (25.8%)	78 (15.4%)	16 (16.8%)	
		Other	93 (5.1%)	64 (5.3%)	26 (5.1%)	3 (3.2%)	
		Private	419 (23.2%)	320 (26.6%)	82 (16.1%)	17 (17.9%)	
		Uninsured	188 (10.4%)	123 (10.2%)	61 (12.0%)	4 (4.2%)	
Injury severity score			8.9 ± 6.9	8.7 ± 6.8	9.0 ± 6.9	10.7 ± 8.4	.024
Mechanism of Injury							< .001
	Stab		86 (4.8%)	34 (2.8%)	37 (7.3%)	15 (15.8%)	
	Fall		720 (39.8%)	515 (42.8%)	174 (34.3%)	31 (32.6%)	
	Firearm		86 (4.8%)	42 (3.5%)	35 (6.9%)	9 (9.5%)	
	Motor vehicle accident		567 (31.4%)	411 (34.1%)	134 (26.4%)	22 (23.2%)	
	Other		112 (6.2%)	67 (5.6%)	36 (7.1%)	9 (9.5%)	
	Pedestrian/bike		37 (2.0%)	23 (1.9%)	14 (2.8%)	0	
	Struck by or against		167 (9.2%)	91 (7.6%)	70 (13.8%)	6 (6.3%)	
	Rec vehicle		32 (1.8%)	21 (1.7%)	8 (1.6%)	3 (3.2%)	
Drugs/alcohol			466 (25.8%)	139 (11.5%)	274 (53.9%)	53 (55.8%)	< .001
Hospital length of stay, days			3 (1-5)	2 (1-5)	3 (1-5)	6 (3-13)	< .001
ICU admission			745 (41.2%)	458 (38.0%)	231 (45.5%)	56 (58.9%)	< .001
ICU length of stay, days			3 (2-6)	3 (2-5)	3 (2-5)	6 (3-10.8)	< .001
Ventilated			212 (11.7%)	100 (8.3%)	78 (15.4%)	34 (35.8%)	< .001
Ventilated days			3 (2-5.8)	3 (2-8)	2 (2-4.3)	3.5 (2-6)	.216
Median hospital cost, $ thousands			18.3 (9.1-18.8)	17.6 (8.8-27.8)	18.7 (9.1-29.3)	26.0 (13.3-51.7)	< .001

The most frequent psychiatric disorders for the cohort not requiring a consultation were depression (23.6%) and anxiety (16.3%), followed by bipolar (6.2%). The vast majority of patients requiring psychiatric service consultation had an existing psychiatric disorder (n = 87, 91.6%), and 32 (33.7%) of the 95 patients consulted were diagnosed with a new psychiatric disorder. The most common in-patient diagnoses were delirium (n = 14, 14.7%), psychosis (n = 4, 4.2%), and intermittent explosive disorder (n = 3, 3.2%). A total of 24 (1.3%) admissions were due to injuries sustained from self-harm. Self-harm was associated with longer hospital length of stay (7.4 ± 6.7 days vs 4.1 ± 4.4 days, P = .027; Hedges’ g = .73) and increased mean hospital cost ($37,542 ± $31.971 vs $23,674 ± $21,459, P = .045; Hedges’ g = .64).

Patients requiring psychiatric service consultation were the youngest of the three groups (P < .001; eta-squared = .02), had the highest injury severity (P = .024; eta-squared = .004), and the highest incidence of injury sustained by firearm (9.5%) and stabbing (15.8%; P < .001). Of the three groups, patients requiring in-hospital psychiatric consultation had the longest hospital length of stay (P < .001; eta-squared = .04), ICU length of stay (P < .001; eta-squared = .01), were most likely to be ventilated (P < .001), least likely to be discharged to home (P < .001), and had the highest median hospital cost (P < .001; eta-squared = .01).

The correlation between time to psychiatric consult and hospital length of stay bordered moderate to large, r(70) = .50, P < .001. This result along with previous research calling on the need to evaluate the timing of psychiatric involvement led us to explore the impact of early psychiatry consultation on hospital length of stay and cost. Adjusted for injury severity, ICU admission, intubation, and self-harm, early consultation was associated with an average length of stay of 2.9 days less compared to late consultation (P = .021). The same covariates with the addition of hospital length of stay were used in our model predicting hospital costs. In this model, early consultation was associated with an average saving of $7,525 (Table 2). Additional models were also run with the inclusion of psychiatric diagnoses. Above and beyond the results presented in Table 2, specific diagnoses were not associated with cost or length of stay (data not shown).

**Table 2:** Summary of regression models predicting hospital length of stay and cost (n = 1,107).

		Unstandardized beta	Standard error	91% CI for beta	P-value
Dependent variable: Hospital length of stay, days					
	Injury severity score	0.33	0.08	0.18-0.48	< .001>
	ICU admission	3.16	1.55	0.07-6.25	.045
	Ventilated	0.70	1.64	−2.58-3.98	.671
	Self-harm	−1.02	1.35	−3.72-1.68	.451
	Early psychiatric consult	−2.90	1.23	−5.36 to −0.45	.021
Dependent variable: Hospital cost, $					
	Injury severity score	$594	246	101-14,027	.019
	Hospital length of stay	$2,707	366	1,975-3,439	< .001>
	ICU admission	$4,850	4,608	4,364-14,066	.297
	Ventilated	$2,096	4,737	7,376-11,570	.660
	Self-harm	$7,933	3,910	113-15,753	.047
	Early psychiatric consult	$−7,525	3,699	−14,923 to −127	.046

## Discussion

The purpose of this paper was to evaluate hospital resource utilization for trauma patients with psychiatric illnesses. Findings from previous studies have established linkages between psychiatric illness and increased hospital length of stay and cost within the trauma patient population. A study conducted in the year 2000 found 29% of trauma patients had a psychiatric illness and called for future research to investigate the impact of early diagnosis on outcomes as a way to more efficiently provide treatment to this group.

In this study, one-third of our trauma cohort had either a psychiatric diagnosis or received in-hospital psychiatric service consultation, similar to data reported by Zatzick et al. and exceeding the national statistic of incidence in psychiatric illness. Similar to previous research, patients requiring a psychiatric consultation were observed to have a longer hospital length of stay and higher hospital costs. Likely attributed to increased length of stay, we also observed higher ICU admission rates and longer intensive care unit stays. Our study adds to the existing literature by examining the timing of the psychiatry consultation service and its impact on outcomes.

To our knowledge, this is the first study exploring the timing of psychiatric service consultation and its association with hospital length of stay and costs in trauma patients. Our results suggest that hospital costs may be modifiable with earlier psychiatric consultation. The underlying reasons for cost savings associated with early involvement of the psychiatry service, however, remain unestablished. Early initiation of treatment may have contributed to a shorter length of stay thereby impacting cost. Similarly, earlier determination of psychiatric disposition needs may have optimized subsequent discharge planning.

Typical of most trauma centers in the United States, surgeons at our center request psychiatric consultations as needed and based on clinical judgment. The process our surgeons follow to determine the need for a consultation is not a well-documented policy that is strictly adhered to by our providers. Thus, not all patients are seen by the psychiatric service and the timing of consultation varies. For this study, we defined a threshold of early versus late psychiatric consultation based on the day of or the day following hospital admission. This threshold was chosen upon input from our clinical team; however, it should be further evaluated. We recognize that later psychiatric consultation may have been attributable to the patient’s injury burden taking precedence over their psychiatric care as the need for consultation would be unlikely recognized in a heavily sedated patient. Additionally, patient days at risk for experiencing psychiatric symptoms are increased along with hospital length of stay; however, the extent to which patients are sedated should also be considered. Ultimately, psychiatric service consultation beyond our defined early versus late threshold may have been unavoidable and should be further evaluated using a homogenous cohort of trauma patients.

Early psychiatric consultation, however, emerged as a significant predictor of cost and hospital length of stay when controlling for injury severity score, ICU admission, and mechanical ventilation in models consisting of Medicare and Medicaid patients. Although the current study merits further exploration of the mechanism by which psychiatric consultation impacts patient outcomes and the impact of specific diagnoses on outcomes, our results suggest that early consultation when indicated meaningfully impacts inpatient trauma care. Cost savings and reduction in length of stay may be achieved with early involvement of the psychiatry service in patients with concomitant or suspected psychiatric diagnoses and should be considered for protocolized implementation into trauma care.

## Conclusions

Previous studies have established an association between psychiatric illness and outcome in the trauma patient population. In this study, we replicated findings suggesting psychiatric consultation overall was associated with increased hospital length of stay and cost. In addition, the timing of psychiatric service consultation emerged as a significant predictor with early in-hospital psychiatric service consultation associated with significant savings in hospital length of stay and cost. The optimization of patient care continues to be crucial to the fiscal health of trauma centers. Future research should be conducted emphasizing an integrative approach of the psychiatric and trauma services such that all trauma patients are provided a psychiatric consultation.

## References

[1] (2021). Prevalence of any mental illness. https://www.nimh.nih.gov/health/statistics/mental-illness.shtml.

[2] Townsend LL, Esquivel MM, Uribe-Leitz T (2017). The prevalence of psychiatric diagnoses and associated mortality in hospitalized US trauma patients. J Surg Res.

[3] McAninch J, Greene C, Sorkin JD, Lavoie MC, Smith GS (2014). Higher psychological distress is associated with unintentional injuries in US adults. Inj Prev.

[4] Falsgraf E, Inaba K, de Roulet A (2017). Outcomes after traumatic injury in patients with preexisting psychiatric illness. J Trauma Acute Care Surg.

[5] Warnack E, Choi BH, DiMaggio C, Frangos S, Bukur M, Marshall G (2018). Patients with psychiatric disorders require greater health-care resources after injury. Am Surg.

[6] Clous EA, Beerthuizen KC, Ponsen KJ, Luitse JS, Olff M, Goslings JC (2017). Trauma and psychiatric disorders: a systematic review. J Trauma Acute Care Surg.

[7] Gribben JL, Ilonzo N, Neifert S, Hubert M, Leitman IM (2019). Characteristics and outcomes of abdominal and pelvic trauma patients with psychiatric illness. J Surg Res.

[8] Zatzick DF, Kang SM, Kim SY (2000). Patients with recognized psychiatric disorders in trauma surgery: incidence, inpatient length of stay, and cost. J Trauma.

[9] Lyons JS, Larson DB, Burns BJ, Cope N, Wright S, Hammer JS (1988). Psychiatric co-morbidities and patients with head and spinal cord trauma. Effects on acute hospital care. Gen Hosp Psychiatry.

